# Roles of Methylated DNA Biomarkers in Patients with Colorectal Cancer

**DOI:** 10.1155/2019/2673543

**Published:** 2019-03-03

**Authors:** Zhiyao Ma, Marissa Williams, Yuen Yee Cheng, Wai K. Leung

**Affiliations:** ^1^Department of Medicine, University of Hong Kong, Queen Mary Hospital, 999077, Hong Kong; ^2^Asbestos Disease Research Institute, Sydney Medical School, University of Sydney, Rhodes, NSW 2139, Australia

## Abstract

Colorectal cancer (CRC) is a leading cancer globally; therefore, early diagnosis and surveillance of this cancer are of paramount importance. Current methods of CRC diagnosis rely heavily on endoscopy or radiological imaging. Noninvasive tests including serum detection of the carcinoembryonic antigen (CEA) and faecal occult blood testing (FOBT) are associated with low sensitivity and specificity, especially at early stages. DNA methylation biomarkers have recently been found to have higher accuracy in CRC detection and enhanced prediction of prognosis and chemotherapy response. The most widely studied biomarker in CRC is methylated septin 9 (SEPT9), which is the only FDA-approved methylation-based biomarker for CRC. Apart from SEPT9, other methylated biomarkers including tachykinin-1 (TAC1), somatostatin (SST), and runt-related transcription factor 3 (RUNX3) have been shown to effectively detect CRC in a multitude of sample types. This review will discuss the performances of various methylated biomarkers used for CRC diagnosis and monitoring, when used alone or in combination.

## 1. Introduction

### 1.1. High Incidence and Mortality of Colorectal Cancer (CRC)

Based on data from the GLOBOCAN study generated in 2012, the global incidence and mortality rates of CRC were shown to increase by 10-fold in a period of 10 years [1]. Specifically, CRC-related mortality is increasing rapidly in many low- and middle-income countries [1]. Furthermore, the incidence of CRC is predicted to continue to increase, especially in developing regions due to changing demographics and aging populations. When comparing the CRC incidence rates between 1988 and 2007 in eight regions globally, it is apparent that this increase is remarkable in both developing and developed countries except in America [2]. While screening for CRC among asymptomatic subjects is important, monitoring for CRC patients after treatment is also crucial. Hence, there is an urgent need to identify more robust early screening and detection biomarkers to facilitate the accurate early diagnosis and surveillance of this common malignancy.

### 1.2. Limitation of Recommended Tests

Although many methods exist for the diagnosis of colorectal cancer, the most accurate diagnostic method is generally considered to be colonoscopy with biopsy. Noninvasive diagnostic tests including blood and stool tests however seem to be more acceptable for screening of asymptomatic subjects as well as CRC patients for surveillance purposes. As yet, most of these noninvasive examinations have relatively low sensitivity and specificity, and false positive or negative results are not uncommon. Carcinoembryonic antigen (CEA) is the most widely used blood glycoprotein marker for CRC, particularly for monitoring of treatment response and surveillance. The American Society of Clinical Oncology has recommended testing of CEA every 3 months for at least 3 years following tumour resection in stages II and III CRC, while the European Group on Tumour Markers (EGTM) recommends testing for those who may receive liver resection or systemic treatment in a frequency of 2-3 months [3, 4]. However, a growing number of studies have casted doubt upon the role of using serum CEA in monitoring CRC recurrence due to arbitrary thresholds used to depict the presence of disease in different studies [5–7]. Shinkins et al. reviewed 52 studies including 9,719 participants to determine the best CEA cut-off threshold, and all three selected thresholds were found to be unsatisfied. It was determined that threshold values of 2.5 *μ*g/l or 5 *μ*g/l produced many false positives (up to 20%), while values of 5 *μ*g/l or 10 *μ*g/l would result in nearly one-third of recurrences being left undiagnosed [6], deeming CEA as an unsatisfactory measure of CRC detection as alluded in other studies [8].

In regard to stool testing, faecal occult blood testing (FOBT) has been recommended for CRC screening in people 50 years or older by EGTM [4]. Unfortunately, FOBT was observed to have lower sensitivity in the proximal colon (71.2%; 95% CI: 61.3–79.4%) than distal colon (80.1%; 95% CI: 70.9–87.0%) [9]. Hence, it is necessary to discover more robust biomarkers for CRC screening and monitoring.

### 1.3. Methylated DNA Biomarkers

Discovery of epigenetic alterations in body fluids is an innovative alternative method of biomarker detection, with the advantages of stability, high frequency of positive detection, and noninvasive accessibility [10]. Among all studied epigenetic biomarkers, DNA methylation is the most frequently examined in various cancers, including CRC [11]. Methylated DNA biomarkers detected in CRC tissue, blood, and stool samples have been increasingly studied in recent years, but in many instances, the significance of their alterations in terms of functionality and biomarker value has not been properly characterized. Many studies have highlighted the potential of methylated DNA biomarkers for CRC detection and monitoring. Recently, methylated septin 9 (SEPT9) has been approved by the U.S. Food and Drug Administration (FDA) for screening of CRC [12]. Moreover, an increase in SEPT9 methylation levels in serum at 1-year follow-up after CRC resection may indicate potential recurrences. On the other hand, other methylated markers may also carry potential prognostic indications such as the methylated tachykinin-1 (TAC1) [13] and insulin-like growth factor binding protein 3 (IGFBP3) [14]. Detection of faecal methylated DNA has also been examined for CRC detection such as the eyes absent homolog 4 (EYA4) that was found to have a sensitivity of 100% (13/13) for CRC detection and 76.9% (27/35) for advanced adenoma, with a specificity of 94.7% (18/19) [15].

In this review, we summarized the performances of methylated markers for the diagnosis and surveillance of CRC (Table 1).

## 2. DNA Methylation Markers

### 2.1. Methylated Septin 9 (SEPT9)

As the only methylated biomarker which has been approved for screening for CRC to date [12], serum SEPT9 has been studied extensively. In a recent systematic review, the second generation of SEPT9 was found to have a high sensitivity (71.1 to 95.6%) and specificity (81.5 to 99%) for CRC detection. When compared to faecal immunochemical test (FIT) in asymptomatic population, SEPT9 had an overall higher sensitivity (75.6% vs. 67.1%) and comparable specificity (90.4% vs. 92.0%) [29]. In our previous study, we found that the sensitivity of SEPT9 was significantly higher than CEA in detecting CRC (75.6% vs. 47.7%, *P* < 0.001) [16]. Monitoring SEPT9 biomarker use in CRC after surgical resection in a prospective cohort study of 150 CRC patients stages I-III, it was found that higher serum SEPT9 levels at 1 year and an increase in methylation from 6 months to 1 year and from preoperation to 1 year were indicative of a lower chance of disease-free survival [13]. Therefore, in addition to its approved diagnostic value, SEPT9 may have prognostic values in CRC.

### 2.2. Twist-Related Protein 1 (TWIST1)

TWIST1 encodes a basic helix-loop-helix transcription factor, which promotes tumour cell invasion and metastasis in multiple human cancers [30]. In 2010, a Japanese study first reported altered TWIST1 methylation levels in different colorectal tissues, with the highest methylation levels in tumour and decreasing levels in colorectal adenoma and normal nontumour colorectal mucosa in CRC patients (median 55.7%, 25.6%, and 0.0%, respectively, *P* < 0.001). Methylated TWIST1 was suggested to be a potential biomarker in early CRC with a high accuracy for tissue detection of 89.6% [17]. Lin et al. examined 353 plasma samples from CRC patients through methylation-specific polymerase chain reaction (MSP) and found that 247 (70.0%) had TWIST1 hypermethylation. However, TWIST1 methylation was not found to have significant prognostic implication, with hazard ratios of 1.06 (*P* = 0.799) and 0.79 (*P* = 0.463), respectively, for univariate and multivariate analyses of disease-free survival [18]. Thus, although methylated TWIST1 is able to differentiate CRC from normal tissues, it may not be a reliable prognostic marker.

### 2.3. Runt-Related Transcription Factor 3 (RUNX3)

RUNX3, a member of the RUNX family, has been shown to participate in various cancer pathways, including cell growth, apoptosis, and angiogenesis. RUNX associates with the Wnt oncogenic and TGF-*β* tumour suppressive pathways to promote CRC development [31]. The role of RUNX3 methylation has also been examined for diagnostic value in CRC in multiple studies [19–22, 32]. Huang et al. determined RUNX3 methylation levels in 30 colorectal cancer tissues and their paired adjacent normal tissues, showing that the RUNX3 methylation levels were significantly higher in tumour than in adjacent tissues (28% vs. 15%, *P* < 0.01) [19]. Shin et al. also observed that tissue RUNX3 hypermethylation had a sensitivity of 32.3% (20/62) and a specificity of 100.0% (0/10) for CRC detection. However, RUNX3 methylation levels were not found to be associated with stage (*P* = 0.307) and differentiation (*P* = 0.179) of tumours, but higher levels were linked with vascular (*P* = 0.006) and lymphatic (*P* = 0.002) invasions and worse prognosis (*P* = 0.038) [20]. In another study, hypermethylated RUNX3 was also detected in 41.5% (27/65) of CRC patients' serum samples [21]. Moreover, it was observed that a higher serum methylation level of RUNX3 was detected in patients with stages III and IV CRC than in healthy controls (*P* = 0.0001). In a 3-year follow-up study after resection of the primary tumour, the preoperative methylated levels of RUNX3 of 52 patients with recurrence were significantly higher than that of 292 patients without recurrence (*P* = 0.0003) [22]. Hence, based on this investigation, RUNX3 not only has potential for CRC diagnosis but also may be useful in predicting CRC recurrence after operation.

### 2.4. Tachykinin-1 (TAC1)

The tachykinins are a family of neuropeptides that share a common carboxyl terminus [33]. TAC1 is a member of this family and is the derivation of substance P and neurokinin A, which influence secretion, motility, and inflammatory reactions in the gastrointestinal tract [33]. The diagnostic potential of detecting and monitoring TAC1 methylation in CRC has been examined in a few studies [23–25]. Mori et al. found that methylated TAC1 was detected in 47% (16/34) of CRC tissue when compared to 12% (2/17) in normal colon mucosa (*P* = 0.01) [23]. Higher serum methylation levels of TAC1 6 months postoperation and an increasement of methylation levels during the first half-year interval were shown to be associated with cancer recurrence (both *P* ≤ 0.001). When compared to serum CEA, the sensitivity of TAC1 for detecting recurrence was higher (58.1% vs. 32.6%, *P* = 0.019) at 6 months postresection and was able to detect CRC clinical recurrence 2.2 months prior to CEA [13]. However, two updated studies both demonstrated conflicting results and concluded that blood TAC1 hypermethylation was not a satisfactory biomarker for survival (*HR* = 1.15, *P* = 0.612 and *HR* = 1.56, *P* = 0.047) [24, 25].

### 2.5. Insulin-Like Growth Factor Binding Protein 3 (IGFBP3)

IGFBP3 is one of the six homologous proteins which has high binding affinity with insulin-like growth factors I and II and can induce apoptosis and affect DNA synthesis [34]. The data on the association between IGFBP3 methylation and CRC however remains controversial. In a study carried out by Perez-Carbonell et al., IGFBP3 had higher diagnostic accuracy (83.0%) than five other markers (miR-137 78.3%, TWIST1 69.3%, SEPT9 65.8%, ALX4 61.6%, and GAS7 37.3%) for CRC, and low methylation levels of IGFBP3 indicated poor survival outcomes (*P* = 0.01). Contrastingly, in another study for stages II and III CRC patients who had received 5-fluorouracil- (5-FU-) based adjuvant chemotherapy, low IGFBP3 methylation levels were associated with longer overall survival (*P* = 0.0007) and disease-free survival (*P* = 0.05). In addition, chemotherapy did not enhance survival in patients with high IGFBP3 methylation levels [14]. Keeping with these findings, Fu et al. showed that the 5-year recurrence-free survival rate in stage II CRC with low methylation IGFBP3 was 3-fold higher than that of cases with high methylation (75.7% vs. 25.0%, respectively). Additionally, high IGFBP3 methylation levels in primary tumour were associated with recurrence (*P* = 0.004) [27]. Yi et al. also found that stage II CRC patients with tumour-methylated IGFBP3 had worse survival than those with unmethylated IGFBP3 (*P* < 0.05), and the former might benefit from adjuvant chemotherapy [26]. Hence, the clinical significance of IGFBP3 methylation levels remains controversial, and more studies are needed to characterize the importance of IGFBP3 as a prognostic marker.

### 2.6. Eyes Absent Homolog 4 (EYA4)

Eyes absent (EYA) is a key regulator of ocular development in Drosophila, and EYA4 belongs to the family of its four homologues [35]. Methylated EYA4 is detectable in a variety of samples from CRC patients including serum, stool, and tumour tissue. Kim et al. detected tissue EYA4 methylation in CRC, paired normal colonic mucosae, and advanced adenoma, with positive rates of 93.5% (43/46), 32.6% (15/46), and 50.7% (36/71), respectively. They had also detected EYA4 methylation in stool samples and obtained a sensitivity of 83.3% (95% confidence interval (CI): 0.70-0.91) and a specificity of 94.7% (95% CI: 0.75-0.99) for diagnosing CRC and advanced adenoma [15]. Liu et al. detected serum methylated EYA4 in 26 Chinese patients with stage I CRC, with a sensitivity of 57.7% and a specificity of at least 90% [28]. However, serum EYA4 methylation was found to have no significant association with CRC recurrence or cancer-specific survival. EYA4 methylation in blood or tissue samples also had no association with radiological treatment for metastatic CRC [13, 36].

### 2.7. Somatostatin (SST)

SST, a peptide synthesized in multiple tissues including the gastrointestinal tract, could act as a neurotransmitter or an inhibitory hormone [37]. Methylated SST was detected in 88% (30/34) of primary colorectal tumours, which was significantly higher than that in normal colon mucosae (*P* < 0.001) [23]. The level of SST methylation was also found to be higher in stage I CRC patients than in normal controls (*P* = 0.037). Serum methylated SST was significantly associated with cancer-specific survival among all other detected markers tested in the same study (TAC1, MAL, SEPT9, NELL1, CRABP1, EYA4, and CEA) at the preoperative time point, while its methylation status after operation had no value on prognosis [13, 28]. On the other hand, Liu et al. showed that the high serum SST methylation group had higher cancer recurrence after surgery than the low methylation group (38.7% vs. 18.7%, *P* = 0.005), and cancer-specific survival and disease-free survival were both longer in the latter as determined by univariate or multivariate Cox analysis (all *P* < 0.05) [24].

### 2.8. Combined Methylation Markers

Combining multiple methylated biomarkers may increase the diagnostic and prognostic accuracies for CRC. Perez-Carbonell et al. compared the accuracy of combined tissue methylated markers (TWIST1, IGFBP3, and miR-137) for the diagnosis of CRC. They found that the combined methylation markers increased the diagnostic accuracy to 92.0%, followed by miR-137+IGFBP3 (86.0%), IGFBP3 (83.0%), TWIST1+IGFBP3 (82.7%), TWIST1+miR-137 (78.5%), miR-137 (78.3%), and TWIST1 (69.3%) [14]. Liu et al. demonstrated that the combination of serum methylation markers (TAC1 and EYA4) had a sensitivity level of 84.6% and a specificity of 80.8% for detecting CRC, while a combination of serum TAC1 and SEPT9 displayed an increased level of specificity of 92.3% with a sensitivity of 73.1% [28]. The combination of serum methylated TAC1, SEPT9, and NELL1 could also depict a higher cancer-specific death risk after CRC surgical resection (*P* = 0.001) than any single marker at 6 months but not after 1 year postsurgery [13].

CpG island methylator phenotype (CIMP) is a combination of methylation markers for diagnosis which has been studied extensively [38–43]. Early in 2009, Ogino et al. found that stages I-IV CRC patients who had CIMP-high tumour (defined as ≥6 of the 8 promoters positive: CACNA1G, CDKN2A, CRABP1, IGF2, MLH1, NEUROG1, RUNX3, and SOCS1) experienced significantly lower cancer-specific mortality [38]. However, some studies showed an inverse association between CIMP status and CRC prognosis. Vedeld et al. observed that CIMP-positive (≥3/5 promoters positive: CACNA1G, IGF2, NEUROG1, RUNX3, and SOCS1) CRC cases were significantly associated with a shorter recurrence time and worse overall survival after surgery [39]. Cha et al. also showed that the overall survival of metastatic CRC was longer in the lower-methylation group when eight markers were tested, with median survival of 9.77, 22.2, and 35.7 months for high CIMP (≥5), low CIMP (1-4), and CIMP-negative (0) groups, respectively (*P* < 0.001) [42]. The CIMP phenotype has also been monitored in response to chemotherapeutic agents with conflicting results. In some studies, improved prognosis was obtained for CRC patients with negative or low CIMP who received chemotherapy including 5-FU or/and oxaliplatin, while others found that positive or high CIMP was associated with better outcome after chemotherapy [40, 42]. Furthermore, some studies found no association between CIMP status with CRC chemotherapy [41, 43]. These conflicting results could be partly attributed to the inconsistent definition of CIMP in different studies.

### 2.9. Combination of DNA Methylation with CEA or FIT

Similar to the use of combined methylated markers, the combination of CEA or FIT with methylation markers could also improve the sensitivity of these tests for CRC. Suehiro et al. recently studied the diagnostic role of detecting TWIST1 methylation in faeces. The combined faecal TWIST1 methylation status tested together with FIT increased sensitivity to 82.4%, which was compared to the sensitivities of 47.1% and 41.2% when the test was used alone [44]. When compared to the use of a single marker, the combination of both CEA and CA19-9 with RUNX3 methylation provided higher sensitivity in the detection of CRC and did not reduce specificity [21].

These studies highlight the advantages of employing a combination of biomarkers to detect CRC. However, more studies are required to determine precisely which biomarkers should be selected and the optimal number of markers to be effective when considering the cost, complexity, and performance of these markers.

## 3. Current Issues Related to Detection of DNA Methylation

### 3.1. Tumour Characteristics

Methylated gene biomarkers are usually detected in tumours of higher staging, particularly in blood samples. Nishio et al. found that the average methylation ratio of RUNX3 in serum and tumour tissue increased with higher tumour stages (*P* = 0.0466 and *P* = 0.0018, respectively) [22]. By studying gene methylation distributions of 353 plasma samples from CRC patients of different tumour stages, Lin et al. found that AGBL4 (ATP/GTP binding protein-like 4) and FLI1 (friend leukaemia integration 1 transcription factor) methylation had the highest sensitivities in stage IV (77.8% and 81.0%, respectively) and lowest sensitivities in stage II (58.6% and 52.9%, respectively) [18]. Moreover, serum SST methylation was found to be a predictive value of cancer-specific survival in stage III patients as determined by multivariate Cox analysis (*HR* = 2.52, *P* = 0.045) but was not found to be significant in patients with stage II cancers (*P* = 0.08) [24].

Most studies showed that DNA methylation levels were usually associated with right-sided CRC. In a study conducted by Nishio et al., average tissue methylation levels of RUNX3 in the proximal colon were higher than that in the distal colon (*P* = 0.0054), but differential levels of sensitivity were not observed in serum methylation (*P* = 0.2551) [22]. Fu et al. observed higher IGFBP3 methylation in right-sided CRC as compared to left-sided CRC (*P* < 0.001) [27]. Additionally, Vedeld et al. found that CIMP-positive tumours were more frequently present in proximal CRC [39].

When comparing methylation levels of TAC1, SEPT9, NELL1, and SST in tumours and paired serum, methylation levels were consistently higher in tumours (*P* < 0.05) while there was no significant association of methylation statuses between the two sample types [13, 24].

### 3.2. Methods to Detect Methylation

**Figure 1:**
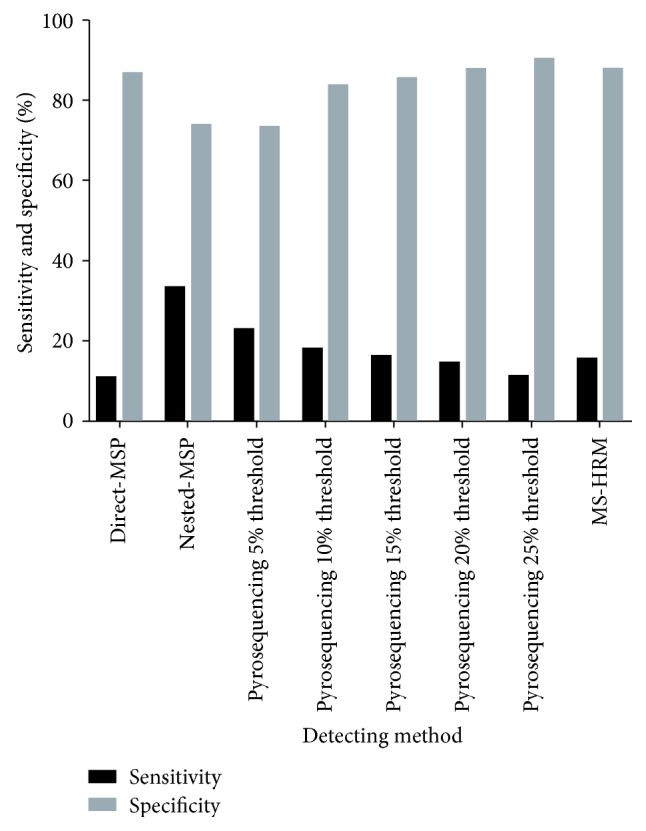
Clinical sensitivity and specificity for methylation detection through different methods. Sensitivity was calculated as *a*/(*a* + *b*). Specificity was calculated as *d*/(*c* + *d*). MSP: methylation-specific PCR; MS-HRM: methylation-sensitive high-resolution melting.

Different methods to detect DNA methylation may alter the perceived methylation levels. Draht et al. compared four different methods in detecting CpG island methylation for 241 stage II CRC patients and found that nested-MSP had the highest sensitivity (33.1%) and was more effective compared to direct-MSP (10.7%), while pyrosequencing of 25% threshold obtained the best clinical specificity (90.2%), followed by methylation-sensitive high-resolution melting (87.7%) (Figure 1). However, there was no significant difference found in terms of prognostic implications when comparing different methods (*P* > 0.05) [45]. Recent studies have shown that droplet digital polymerase chain reaction (ddPCR) has advantages of increased precision, accuracy, and technical simplicity in comparison to conventional quantitative MSP, while high-resolution melting analysis was better than ddPCR in genotyping small deletion and insertion polymorphisms [46, 47]. However, results on their determination of methylated DNA from CRC patients are lacking.

### 3.3. Sample Type and Timing

In a recent study analyzing the SEPT9 methylation status of 9 CRC patients, plasma samples were collected at four separate times (06:00, 12:00, 18:00, and 24:00) in a day for testing. The results showed higher methylation values at 24:00 than any other time points (100% vs. 77.7%), and two stage I cases only had positive SEPT9 methylation at 24:00 [48]. Another earlier study explored the variation of DNA methylations of normal individuals after collecting 9 blood samples from each person at 3-hour intervals during 24 hours and discovered an increase of DNA methylation from 23:00 to 02:00 followed by a decline in levels at 08:00 (*P* = 0.021). This was opposite to the trend observed in contemporaneous homocysteine levels, an amino acid which participates in the one-carbon metabolic pathway of DNA methylation for CRC, where there was no significant variation of its methylation status in the daytime [49, 50]. It may therefore be concluded that circadian variation of DNA methylation exists in CRC, which is probably related to cellular metabolic pathways. Larger-scale research of various methylated DNA biomarkers should be conducted to confirm these time-dependent observations.

## 4. Conclusion

Methylated genes have been shown to have potential in diagnosing, monitoring, and predicting chemotherapy response in CRC. The detection of methylated markers in serum/plasma or faecal samples represents a new, noninvasive method for cancer detection as well as a tool for monitoring during treatment. In addition, a combination of methylated markers has demonstrated an improved sensitivity and specificity of detection when compared to the currently used biomarkers CEA, FOBT, or FIT. Some internal and external factors including tumour stage, tumour location, and methylation detection technology can influence the perceived methylation levels; therefore, standardization of sample collection and methylation detection methods is required for clinical implementation in future studies.

## Figures and Tables

**Table 1:** DNA methylation biomarkers in detecting colorectal cancer.

Methylated gene	Sample type	Detecting method	No. of patients	Sensitivity^∗^	No. of controls	Specificity^∗^	*P* value for prognosis^∗∗^	Reference
SEPT9	Blood	qPCR	90	75.6%	NA	NA	NA	[16]
	Serum	qMSP	150	NA	NA	NA	*P* < 0.05	[13]
TWIST1	Tissue	MSP	319	55.7%	215	100%	NA	[17]
	Plasma	MSP	353	70.0%	NA	NA	*P* > 0.1	[18]
RUNX3	Tissue	MSP	30	28%	30	85%	NA	[19]
	Tissue	MSP	62	32.3%	10	100%	*P* = 0.038	[20]
	Serum	MSP	65	41.5%	NA	NA	NA	[21]
	Serum	MSP	344	29%	56	100%	*P* = 0.0003	[22]
	Tissue	MSP	119	39%	NA	NA	NA	[22]
TAC1	Tissue	MSP	34	47%	17	88%	NA	[23]
	Serum	qMSP	150	NA	NA	NA	*P* ≤ 0.001	[13]
	Serum	MSP	165	NA	NA	NA	*P* = 0.612	[24]
	Serum	MSP	193	NA	NA	NA	*P* = 0.047	[25]
IGFBP3	Tissue	qMSP	425	44.9%	21	NA	NA	[14]
	Tissue	MSP	147	NA	NA	NA	*P* < 0.05	[26]
	Tissue	MSP	115	NA	NA	NA	*P* = 0.004	[27]
EYA4	Tissue	MSP	46	93.5%	46	67.4%	NA	[15]
	Stool	MSP	13	100%	19	94.7%	NA	[15]
	Serum	qMSP	26	57.7%	26	≥90%	NA	[28]
	Serum	qMSP	150	NA	NA	NA	*P* > 0.05	[13]
SST	Tissue	MSP	34	88%	17	53%	NA	[23]
	Serum	qMSP	150	NA	NA	NA	*P* < 0.05	[13]
	Serum	MSP	165	NA	NA	NA	*P* < 0.05	[24]

^∗^Sensitivity refers to the hypermethylation rates in colorectal cancer samples, while specificity refers to the opposite rates in normal samples. ^∗∗^*P* value for association of DNA hypermethylation with poorer prognosis, including cancer recurrence and reduced survival. SEPT9: methylated septin 9; TWIST1: twist-related protein 1; RUNX3: runt-related transcription factor 3; TAC1: tachykinin-1; IGFBP3: insulin-like growth factor binding protein 3; EYA4: eyes absent homolog 4; qPCR: quantitative polymerase chain reaction; qMSP: quantitative methylation-specific PCR; SST: somatostatin; NA: not available.

## Abbreviations

5-FU: 5-Fluorouracil
AGBL4: ATP/GTP binding protein-like 4
ALX4: Homeobox protein aristaless-like 4
CACNA1G: Calcium voltage-gated channel subunit alpha1 G
CDKN2A (p16): Cyclin-dependent kinase inhibitor 2A
CEA: Carcinoembryonic antigen
CI: Confidence interval
CIMP: CpG island methylator phenotype
CRABP1: Cellular retinoic acid-binding protein 1
CRC: Colorectal cancer
ddPCR: Droplet digital polymerase chain reaction
EGTM: European Group on Tumour Markers
EYA4: Eyes absent homolog 4
FDA: U.S. Food and Drug Administration
FIT: Faecal immunochemical test
FLI1: Friend leukaemia integration 1 transcription factor
FOBT: Faecal occult blood testing
GAS7: Growth arrest specific 7
HR: Hazard ratio
IGF2: Insulin-like growth factor 2
IGFBP3: Insulin-like growth factor binding protein 3
MAL: Myelin and lymphocyte protein
MLH1: MutL homolog 1
MSP: Methylation-specific polymerase chain reaction
NELL1: NEL-like protein 1
NEUROG1: Neurogenin-1
RUNX3: Runt-related transcription factor 3
SEPT9: Methylated septin 9 DNA
SOCS1: Suppressor of cytokine signaling 1
SST: Somatostatin
TAC1: Tachykinin-1
TWIST1: Twist-related protein 1.
